# Efficacy and Safety of Polymer-Free Ultrathin Strut Sirolimus-Probucol Coated Drug-Eluting Stents for Chronic Total Occlusions: Insights from the Coroflex ISAR 2000 Worldwide Registry

**DOI:** 10.1155/2018/8053168

**Published:** 2018-03-01

**Authors:** Ahmad Syadi Mahmood Zuhdi, Florian Krackhardt, Matthias W. Waliszewski, Muhammad Dzafir Ismail, Michael Boxberger, Wan Azman Wan Ahmad

**Affiliations:** ^1^Cardiology Unit, University Malaya Medical Centre, 59100 Kuala Lumpur, Malaysia; ^2^Department of Cardiology and Internal Medicine, Charité–Universitäts Medizin Berlin, Campus Virchow, Berlin, Germany; ^3^Medical Scientific Affairs, B. Braun Melsungen AG, Berlin, Germany

## Abstract

**Objective:**

Coronary revascularization in chronic total occlusion (CTO) is associated with improved clinical outcomes. The choice of the coronary stent is crucial in maintaining long-term vessel patency after CTO revascularization. We investigated the efficacy and safety of polymer-free ultrathin strut sirolimus-probucol coated drug-eluting stents (PF-SES) for CTO lesions.

**Methods:**

Patients with CTO lesions treated with PF-SES were identified from the prospective multicenter international ISAR 2000 registry. The primary endpoint was clinically driven target lesion revascularization (TLR) at 9 months. Secondary endpoints were 9-month major adverse cardiac events (death, myocardial infarction, or TLR) (MACE) and the occurrence of stent thrombosis.

**Results:**

A total of 111 patients with CTO lesions (*n*=127) were available for analysis. The 9-month clinical follow-up rate was 91%. The mean reference vessel diameter and lesion length were 2.76 mm ± 0.40 and 26.8 mm ± 13.1, respectively. The overall DAPT duration was 9.7 ± 2.8 months. Only one (1%) in-hospital MI was reported. The TLR and MACE rates at 9 months were 2% (2/101) and 5.9% (6/101), respectively. The 9-month accumulated rates of definite or probable stent thrombosis was 0% (0/101).

**Conclusion:**

Revascularizations for CTO with PF-SES are associated with low rates of TLR and MACE at 9 months with no stent thrombosis. These initial findings need to be compared with results of other new generation DES of larger studies.

## 1. Introduction

The prevalence of chronic total occlusion (CTO) is reported to be around 18.4% among patients with significant coronary artery disease [1]. Of these CTO patients, only 36% underwent percutaneous revascularization [1]. The lack of predictability in terms of procedural success and vessel patency post percutaneous coronary interventions (PCI) have made CTO revascularization not as attractive as non-total occlusive lesions. Furthermore, the failure rate of CTO revascularization is reported to be around 40% [2, 3]. However, the development of new CTO guidewires and techniques together with the use of new generation DES in CTO revascularization has increased the procedural success and safety to the level similar to non-CTO revascularization [4–6].

Ultrathin strut polymer-free, sirolimus-probucol coated drug-eluting stents (PF-SES) are safe and effective in large scale all-comers population with low rate of target lesion revascularization (TLR) [7]. The polymer-free matrix of these stents consists of sirolimus and its matrix builder probucol. Their safety and efficacy have been proven similar to the one reported with zotarolimus stents in ISAR-TEST 5 trial [8]. The objective of this study was to assess the safety and efficacy of PF-SES in the treatment of “real-world” de-novo and restenotic CTO lesions.

## 2. Methods

### 2.1. Patient Population and Centers

The ISAR 2000 all-comers registry (http://ClinicalTrials.gov identifier NCT02629575) is a prospective data collection of patients in 26 Asian (South Korea and Malaysia) and 36 European (Czech Republic, France, Germany, Slovakia, and Spain) cardiac centers.

### 2.2. Inclusion and Exclusion Criteria

Patients 18 years or older with stable angina, objective evidence of myocardial ischemia, and acute coronary syndrome (ACS) who met the requirement for PCI [9] were recruited into the international ISAR 2000 all-comers registry. However, for this study, we only selected patients who were treated for CTO lesions (de novo or restenotic). The numbers of stents or treated vessels with reference vessel diameters from 2.0 mm to 4.0 mm were not limited.

### 2.3. Stents, Periprocedure Details, and Medications

PF-SES (Coroflex ISAR, B. Braun Melsungen, Melsungen AG, Germany) were implanted according to each institution's guidelines and in accordance with proper indications for national reimbursement. The polymer-free stent platform consists of a premounted, thin strut (50/60 *μ*m) cobalt-chromium stent whose abluminal surface only is sandblasted to permit a microporous surface for the polymer-free matrix consisting of sirolimus and probucol. Sirolimus with a concentration of 1.2 *μ*g/mm^2^ is available on the abluminal stent surface only. Sirolimus is the active antiproliferative drug whereas the anti-inflammatory probucol is an excipient which controls the release of sirolimus. Probucol mimics the function of a polymer by retarding the sirolimus release.

Vascular accesses via the femoral or radial artery were both permitted with a recommended introducer sheath of at least 5 Fr in diameter. The technique of stent implantation was up to the operators' discretion. Intravenous heparin (70 IU/kg) was given to all patients and supplemented on an as-needed basis. Platelet aggregation inhibitor loading was not mandatory but recommended if possible prior to PCI according to institutional preferences of the cardiac center.

The use of various antiplatelet inhibition agents (6 months or more) such as clopidogrel (75 mg/day), prasugrel (10 mg/day), or ticagrelor (90 mg twice daily) was permissible (as recommended by the treating physician) while acetylsalicylic acid 100–325 mg daily was prescribed life-long.

### 2.4. Data Collection

To handle the wealth of the data, an electronic data capture system was used. This database was previously used in prior large scale unselected patient cohorts [10, 11] and also used for this assessment [7]. The national principal investigators in each country verified the accuracy of the data when routinely performed web-based plausibility checks indicated any discrepancies. To assure the data quality, automatic queries were sent directly to the investigators.

### 2.5. Endpoints and Definitions

The primary endpoint was the 9-month clinically driven target lesion revascularization (TLR) rate, whereas secondary endpoints were the 9-month major adverse cardiac events (MACE) rate, the in-hospital MACE rate, and the corresponding rates of myocardial infarction (MI) and TLR (coronary artery bypass grafting and percutaneous coronary intervention). Cardiac death was only defined in-hospital, whereas the all-cause death rate was used to define MACE at 9 months (MI, TLR, in-hospital cardiac death, and all deaths post discharge). The Academic Research Consortium (ARC) criteria were used to define acute/subacute stent thrombosis [12].

For simplicity reason and based on a previous protocol [10], a glomerular filtration rate (GFR) of <90 mL/min/1.73 m^2^ with a cut-off GFR rate for mandatory dialysis of <15 mL/min/1.73 m^2^ was used to define renal insufficiency. The angulation criterion of >45° was used to define severe vessel tortuosity.

### 2.6. Statistical Analysis

The statistical methods were also reported elsewhere [7]. The two-sided Fisher's exact and chi-squared tests were used for categorical variables. In case the Shapiro–Wilk test revealed a strong deviation from a normal distribution, the Mann–Whitney test was used instead of the unpaired *t*-test. A cut-off *p* value of 0.05 was considered statistically significant. Statistical analyses were conducted with SPSS V. 24.0 (IBM Munich, Germany). The biometric estimate in the original study [7] was calculated with nQuery/nTerim V.2.0 Advisor (Statistical Solutions Ltd, Cork, Ireland).

### 2.7. Ethical Approval

Ethics committees of all participating centers approved the study protocol prior to patient recruitment. In France, this noninterventional study was nationally approved by Comité Consultative sur le Traitement de I'Information en matière de Recherche dans le domaine de la Santé (CTIRS dossier number 14.613) and the Commission Nationale de I'informatique et des Libertés (CNIL, demande d'autorisation number 915019).

## 3. Results

### 3.1. Patient Characteristics

A total of 111 patients with CTO (Table 1) were enrolled in the international ISAR 2000 all-comers registry which had an overall recruitment of 2877 patients [7]. This amounted to 3.6% (111/2977) of the overall patient cohort. They were stratified into two groups: patients with lesions < 25 mm and ≥25 mm in length. Patient baseline characteristics are described in Table 1. Mean age for the cohort with CTO was 64.9 years. Diabetes mellitus was present in 45.0% (50/111) of patients who were predominantly male (73.9%, 82/111). The rate of acute coronary syndrome (ACS) in CTO cohort; STEMI and NSTEMI were 8.1% (9/111) and 12.6% (14/111), respectively. In general, the two groups were not significantly different in terms of their baseline characteristics except for higher rate of hypertension in the ≥25 mm lesion length group (79.0% versus 61.2%, *p*=0.040).

### 3.2. Lesion Characteristics and Procedural Data

There were 127 CTO lesions (Table 2) which were treated with PF-SES which constituted 3.9% (127/3254) of the total number of lesions in ISAR 2000 all-comers registry [7]. Revascularizations in the right coronary artery (RCA) were most common as compared to the left anterior descending (LAD) and left circumflex artery (LCx), that is, 43.3% versus 29.1% versus 27.6% (*p*=0.005). Of note were the significant differences in lesion characteristics between the short and the long lesion length groups. Diffuse disease (55.2% versus 73.9%, *p*=0.027), in-stent restenosis (1.7% versus 15.9%, *p*=0.006) and B2/C morphologies (89.7% versus 100.0%, *p*=0.006) were more frequent in lesions ≥ 25 mm in length. The average lesion lengths were 16.9 mm versus 35.2 mm, and the average DES lengths were 20.3 mm versus 36.9 mm. The overall technical success rate of PF-SES implantations in CTO lesions was high with 99.2% with no significant differences between groups (*p*=0.357).

### 3.3. PeriProcedural Medications

Preprocedural antiplatelet therapy (Tables 3 and 4) did not differ between the two groups (Table 3). Clopidogrel remained the most preferred P2Y12 inhibitor to combine with aspirin as preprocedural dual antiplatelet therapy (DAPT) regime. The same DAPT distribution consisting of 55–60% clopidogrel, 15% prasugrel, and 10–15% ticagrelor was documented in both subgroups (Table 3). The average duration of DAPT duration was 9.7 months without differences between the groups (Table 4). Most of the patients were treated with DAPT for at least 6 months. There was only one patient exposed to oral anticoagulant in this cohort.

### 3.4. Clinical Outcomes

At 9 months, there were two patients (2%, 2/101) with TLR (primary endpoint). The rates of acute myocardial infarction (MI) and all-cause death at 9 months were 2% (2/101) and 3% (3/101), respectively. Hence, the composite MACE rate at 9 months was 5.9% (6/101). The in-hospital follow-up was event free except for one acute MI (1%, 1/111). There seems to be a higher 9-month MACE rate for the >25 mm lesion group, but the difference did not reach statistical significance (*p*=0.536). There was no report of acute, subacute, or late stent thrombosis (Table 5).

## 4. Discussions

Despite the conflicting data regarding the clinical benefit [3, 4], CTO revascularizations are associated with better clinical outcomes as those in patients with low ejection fraction or ischemic burden [13–15]. Data on long-term outcomes also show lower mortality in patients with successful CTO PCI [16–18].

Unfortunately, due to the technical complexity, relatively lower procedure success rates, and higher periprocedural complications [2, 3], CTO-PCI from the outset is not as appealing as PCI in less complex coronary lesions. Predictors of unsuccessful PCI are identified to be absence of tapered stump structure, TIMI flow grade 0 pre-PCI, high serum creatinine concentrations, and lesion length [19]. However, with the ever-improving PCI techniques and dedicated devices, CTO-PCI has seen significant improvement in terms of procedural success [4–6].

For patients to benefit long term and to justify the risk of CTO-PCI, to maintain the target vessel patency is important. A large CTO registry of 800 patients with 6- to 9-month angiographic follow-up reported a reocclusion rate of 7.5% [20]. This study demonstrated the importance of the choice of stents used in maintaining vessel patency. The use of DES in the registry dramatically reduced both reocclusion and nonocclusive angiographic restenosis compared to BMS [21]. The overall restenosis rate of DES in CTO was 11% [21]. A randomized controlled trial also reported that there is a 55% relative risk reduction (RRR) in MACE with the use of DES compared to BMS in CTO PCI [22].

Our study described the outcomes of CTO-PCI from “real-world” data using polymer-free ultrathin strut sirolimus-probucol coated drug-eluting stents (PF-SES). CTO-PCIs in this study were conducted in various centers with a broad spectrum of operator experience and revascularization techniques. Overall, the results of this study reflect the unrestricted, day-to-day practice from various European and Asian centers in the treatment of CTO.

The outcome of CTO lesions stented with PF-SES from this study is encouraging. The overall 9-month TLR rate of both <25 mm and >25 mm CTO lesions is low at around 2%. We did not observe any acute, subacute, or late stent thrombosis in this study. The MACE rate at 9-month follow-up is also favorably low at 5.9% (4.3% for lesions < 25 mm and 7.3% for lesions > 25 mm, *p*=0.358). By crude comparison, the EXPERT-CTO trial of XIENCE reported a clinically driven TLR rate of 6.3% and 0.9% subacute stent thrombosis and 0.5% late probable stent thrombosis at one year [23].

## 5. Conclusion

The results support the use of PF-SES as an efficacious and safe therapeutic option in the treatment of CTO. The result at least for short term (9 months) is promising, and a longer follow-up would be of interest to determine if PF-SES angioplasty is able to maintain vessel patency in the long-term (>1 year).

### 5.1. Strengths and Limitations of This Study

The CTO cohort in this study was extracted from the largest all-comers registry under routine use of PF-SES, which can be safely and effectively implanted with favorable rates of TLR and MACE. The data suggest that the use of PF-SES in high-risk patients with complex lesions (diabetes mellitus, ACS, diffuse disease, and CTO) is feasible. However, since this is a subgroup analysis from a “real-world” observational study, data collection and monitoring may not have been as stringent as in randomized control trials with the possibility of some underreporting of events. Although the number of patients reported in this assessment was small, the results nevertheless described the potential of PF-SES performance in the specific cohort of CTO patients. It also would have been useful to provide more details on the chronic kidney disease stages relative to their glomerular filtration rates, which was not done in this assessment. Likewise, the use of the J-SCORE and/or the time course of the occlusion along with the lesion crossing (antegrade/retrograde) would have helped to provide additional details of the performed CTO recanalization.

## Figures and Tables

**Table 1:** Patient demographics.

Variable		Patients, *n* (%)	<25 mm lesion length, *n* (%)	≥25 mm lesion length, *n* (%)	*p* value
Number of patients		111 (100%)	49 (44.1%)	62 (55.8%)	—
Number of lesions		127 (100%)	58 (45.7%)	69 (54.3%)	—
Number of DES used		160 (100%)	65 (40.6%)	95 (59.4%)	—
Age (years)		64.9 ± 11.6	64.8 ± 12.3	65.0 ± 11.0	0.942
Male gender		82 (73.9%)	35 (71.4%)	47 (75.8%)	0.602
Diabetes		50 (45.0%)	19 (38.8%)	31 (50.0%)	0.238
Hypertension		79 (71.2%)	30 (61.2%)	49 (79.0%)	0.040
Renal insufficiency		6 (5.4%)	3 (6.1%)	3 (4.8%)	0.766
Dialysis dependence		1 (0.9%)	0 (0.0%)	1 (1.6%)	0.372
STEMI		9 (8.1%)	2 (4.1%)	7 (11.3%)	0.257
NSTEMI		14 (12.6%)	8 (16.3%)	6 (9.7%)	
Region	Europe	71 (64.0%)	29 (59.2%)	42 (67.7%)	0.351
	Asia	40 (36.0%)	20 (40.8%)	20 (32.3%)	

**Table 2:** Lesion characteristics and procedural data.

Variable		Patients, *n* (%)	<25 mm lesion length, *n* (%)	≥25 mm lesion length, *n* (%)	*p* value
Number of lesions		127 (100%)	58 (45.7%)	69 (54.3%)	—
Target vessel	LAD	37 (29.1%)	15 (25.9%)	22 (31.9%)	0.005
	LCx	35 (27.6%)	24 (41.4%)	11 (15.9%)	
	RCA	55 (43.3%)	19 (32.8%)	36 (52.2%)	
	Graft	0 (0.0%)	0 (0.0%)	0 (0.0%)	
Multivessel disease	1-vessel	103 (81.1%)	46 (79.3%)	57 (82.6%)	0.298
	2-vessels	22 (17.3%)	10 (17.2%)	12 (17.4%)	
	3-vessels	2 (1.6%)	2 (3.4%)	0 (0.0%)	
Thrombus burden		10 (7.9%)	5 (8.6%)	5 (7.2%)	0.775
Diffuse vessel disease		83 (65.4%)	32 (55.2%)	51 (73.9%)	0.027
Calcification		54 (42.4%)	24 (41.4%)	30 (43.5%)	0.812
Ostial lesion		9 (7.1%)	5 (8.6%)	4 (5.8%)	0.537
Bifurcations		8 (6.3%)	5 (8.6%)	3 (4.3%)	0.324
In-stent restenosis		12 (9.4%)	1 (1.7%)	11 (15.9%)	0.006
Severe tortuosity		17 (13.4%)	7 (12.1%)	10 (14.5%)	0.689
AHA/ACC type B2/C lesion		121 (95.3%)	52 (89.7%)	69 (100.0%)	0.006
Reference diameter (mm)		2.76 ± 0.40	2.7 ± 0.4	2.8 ± 0.4	0.059
Lesion length (mm)		26.8 ± 13.1	16.9 ± 4.5	35.2 ± 11.9	<0.001
DESs used		160 (100%)	65 (40.6%)	95 (59.4%)	—
DES per patient		1.8 ± 1.3	1.4 ± 1.2	2.1 ± 1.4	0.008
DES diameter (mm)		2.7 ± 0.4	2.7 ± 0.4	2.7 ± 0.4	0.690
DES length (mm)		29.4 ± 15.8	20.3 ± 8.4	36.9 ± 16.5	<0.001
DES inflation pressure (atm)		14.3 ± 3.4	14.5 ± 3.2	14.2 ± 3.5	0.638
Final result (% stenosis)		4.9 ± 11.3	5.7 ± 14.6	4.3 ± 7.7	0.478
Overall technical success per lesion		126 (99.2%)	58 (100.0%)	68 (98.6%)	0.357

**Table 3:** Periprocedural drug therapy.

Drug type		Drug	Patients, *n* (%)	<25 mm lesion length, *n* (%)	≥25 mm lesion length, *n* (%)	*p* value
Pre-PCI	Antiplatelet therapy (APT)	Clopidogrel	59 (53.9%)	29 (59.2%)	30 (48.4%)	0.391
		Prasugrel	18 (16.2%)	7 (14.3%)	11 (17.7%)	
		Ticagrelor	12 (10.8%)	7 (14.3%)	5 (8.1%)	
		Ticlopidine	1 (0.9%)	0 (0.0%)	1 (1.6%)	
		Aspirin only	10 (9.0%)	2 (4.1%)	8 (12.9%)	
		No preloading	11 (9.9%)	4 (8.2%)	7 (11.3%)	
	Oral anticoagulation (OAC)	All OAC	1 (0.9%)	1 (2.0%)	0 (0.0%)	0.258
		Vitamin K antagonist (VKA)	0 (0.0%)	0 (0.0%)	0 (0.0%)	0.258
		New oral anticoagulation (NOAC)	1 (0.9%)	1 (2.0%)	0 (0.0%)	
Post-PCI	Antiplatelet therapy (APT)	Clopidogrel	92 (82.9%)	40 (81.6%)	52 (83.9%)	0.167
		Prasugrel	4 (3.6%)	0 (0.0%)	4 (6.5%)	
		Ticagrelor	12 (10.8%)	4 (16.3%)	4 (6.5%)	
		Aspirin only	1 (0.9%)	0 (0.0%)	1 (0.9%)	
		Unknown	2 (1.8%)	1 (2.0%)	1 (0.9%)	

**Table 4:** Recommended duration of dual antiplatelet therapy during follow-up.

Variable	Patients, *n* (%)	<25 mm lesion length, *n* (%)	≥25 mm lesion length, *n* (%)	*p* value
Number of patients	111 (100%)	49 (44.1%)	62 (55.8%)	—
DAPT duration in months	9.7 ± 2.8	9.8 ± 2.8	9.6 ± 2.9	0.773
1 month	1 (0.9%)	1 (2.0%)	0 (0.0%)	0.355
1–3 months	0 (0.0%)	0 (0.0%)	0 (0.0%)	
3–6 months	0 (0.0%)	0 (0.0%)	0 (0.0%)	
6 months	30 (27.0%)	11 (22.4%)	19 (30.4%)	
>6–12 months	8 (7.2%)	4 (8.2%)	4 (6.5%)	
12 months	50 (45.0%)	20 (40.8%)	30 (48.4%)	
>12 months	0 (0.0%)	0 (0.0%)	0 (0.0%)	
Unknown status	22 (19.8%)	13 (26.5%)	9 (14.5%)	

**Table 5:** Clinical outcomes.

Variable	Patients, *n* (%)	<25 mm lesion length, *n* (%)	≥25 mm lesion length, *n* (%)	*p* value
Number of patients	111 (100%)	49 (44.1%)	62 (55.8%)	—
Patients with clinical follow-up at 9 months or early event	101 (91.0%)	46 (93.6%)	55 (88.7%)	0.345
Follow-up time (months)	8.7 ± 2.5	8.1 ± 2.2	9.2 ± 2.6	0.020
Time to discharge median (IQR) (days)	4.5 ± 16.5	3.2 ± 5.7	5.6 ± 21.7	0.45
In-hospital MACE	1 (1.0%)	0 (0.0%)	1 (1.8%)	0.358
In-hospital TLR	0 (0.0%)	0 (0.0%)	0 (0.0%)	—
In-hospital MI	1 (1.0%)	0 (0.0%)	1 (1.8%)	0.358
In-hospital cardiac death	0 (0.0%)	0 (0.0%)	0 (0.0%)	—
9-month MACE	6 (5.9%)	2 (4.3%)	4 (7.3%)	0.536
9-month TLR (Re-PCI/CABG)	2 (2.0%)	1 (2.2%)	1 (1.8%)	0.898
9-month MI	2 (2.0%)	0 (0.0%)	2 (3.6%)	0.191
9-month death all causes	3 (3.0%)	1 (2.2%)	2 (3.6%)	0.666
9-month accumulated definite/probable stent thrombosis	0 (0.0%)	0 (0.0%)	0 (0.0%)	—
Acute stent thrombosis, ≤24 hours	0 (0.0%)	0 (0.0%)	0 (0.0%)	—
Subacute stent thrombosis, 1–30 days	0 (0.0%)	0 (0.0%)	0 (0.0%)	
Late stent thrombosis, ≥30 days	0 (0.0%)	0 (0.0%)	0 (0.0%)	
BARC 1–5	6 (5.9%)	1 (2.2%)	5 (9.1%)	0.143
BARC 2–5	2 (2.0%)	0 (0.0%)	2 (3.6%)	0.191
BARC 3–5	0 (0.0%)	0 (0.0%)	0 (0.0%)	—
